# Regulation of Id1 expression by epigallocatechin-3-gallate and its effect on the proliferation and apoptosis of poorly differentiated AGS gastric cancer cells

**DOI:** 10.3892/ijo.2013.2043

**Published:** 2013-07-30

**Authors:** JUN MA, MIN SHI, GUANGMING LI, NA WANG, JUE WEI, TING WANG, JIALI MA, YUGANG WANG

**Affiliations:** 1 Departments of Geriatrics, Shanghai Changning Central Hospital, Shanghai 200336;; 2 Gastroenterology, Shanghai Changning Central Hospital, Shanghai 200336;; 3 Department of Gastroenterology, Xinhua Hospital, Shanghai Second Medical University, Shanghai 200092, P.R. China

**Keywords:** apoptosis, epigallocatechin-3-gallate, gastric cancer, Id1, RNAi

## Abstract

We investigated the inhibition of apoptosis and proliferation of poorly differentiated AGS gastric cancer cells by epigallocatechin-3-gallate (EGCG), to establish target genes for regulation by EGCG. The proliferation and apoptosis of AGS gastric cancer cells treated with EGCG were observed by cell counting kit (CCK)-8 and flow cytometry. Differential gene expression in AGS cells treated with EGCG was screened by gene expression microarrays. Id1 gene and protein expression were determined by quantitative PCR and western blot analysis. The effect of Id1 on EGCG-induced apoptosis and cell cycle arrest of AGS cells was verified with RNAi. The proliferation and apoptosis of AGS cells treated with siRNA-Id1 was observed by CCK-8 and flow cytometry. EGCG significantly promoted apoptosis and inhibited the proliferation of AGS cells. The Id1 gene was differentially expressed in AGS cells treated with EGCG, and Id1 mRNA and protein were downregulated in AGS cells treated with EGCG, confirmed by quantitative PCR and western blot analysis. Id1 mRNA and protein were also downregulated in AGS cells treated with siRNA-Id1. The apoptosis and proliferation of AGS cells treated with siRNA-Id1 were similar to those in cells treated with EGCG. EGCG induces apoptosis and inhibits proliferation of poorly differentiated AGS gastric cancer cells, and Id1 may be one of the target genes regulated by EGCG in cancer inhibition.

## Introduction

Gastric cancer has one of the highest incidence rates and mortality worldwide, especially in East Asia ( 1 , 2 ) . More than 400,000 new patients with gastric cancer are diagnosed in China every year. The prevalence and mortality in our country are higher than the world average ( 3 ) . Most gastric cancer is adenocarcinoma, especially poorly differentiated gastric adenocarcinoma, which accounts for approximately 54%. It features high-degree of malignancy, rapid metastasis, and poor prognosis, whereas well-differentiated gastric adenocarcinoma is characterized by slow metastasis and good prognosis. It is important to understand fully the molecular mechanism of poorly differentiated gastric cancer and effectively interfere with such mechanism. The traditional non-target chemotherapy has severe side-effects, therefore, cancer treatment and research has focused on molecular target therapy due to its high selectivity, good efficacy and low incidence of side-effects ( 4 ) .

Tea is rich in polyphenols with strong antioxidant activity ( 5 ) , and has a high level of epigallocatechin-3-gallate (EGCG) ( 6 ) . Research on humans and animal cell models has shown that EGCG has a variety of pharmacological effects, such as strong free-radical scavenging, anti-lipid peroxidation and antiapoptotic, antiviral and antitumor activities ( 7 ) . An imbalance between cell proliferation and apoptosis is one of the primary factors in the occurrence and development of tumors, therefore, proliferation inhibition and apoptosis induction of tumor cells are important methods for prevention and treatment. Many studies have confirmed that EGCG inhibits proliferation of tumor cells and induces apoptosis ( 8 , 9 ) such as lung cancer, nasopharyngeal carcinoma, breast cancer, gastric cancer, colon cancer, prostate cancer, liver cancer, oral cancer, ovarian cancer and other malignant tumors ( 10 – 12 ) , but its molecular mechanism is unknown and needs further study.

In the present study, we observed that EGCG induced apoptosis and inhibited proliferation of poorly differentiated AGS gastric cancer cells. The search for target genes regulated by EGCG verified that the target genes could play a role in apoptosis induction and inhibition of proliferation.

## Materials and methods

### Materials

AGS cells were purchased from the Cell Resource Center of Shanghai Institutes for Biological Sciences, Chinese Academy of Sciences (Shanghai, China), Ham’s F12 medium from HyClone (Logan, UT, USA), trypsin-EDTA solution and fetal bovine serum from Invitrogen (Carlsbad, CA, USA), Cell Counting Kit-8 (CCK-8) from Dojindo (Kumamoto, Japan), EGCG from Sigma (St. Louis, MO, USA), Annexin V-FITC Apoptosis Detection Kit and FACSCalibur Flow Cytometer from BD Pharmingen (San Diego, CA, USA), BioTek micro-plate reader from BioTek (Winooski, VT, USA) with primer designed by Shanghai Sangon Biotech Co. Ltd., Illumina BeadChip HumanHT-12_V4 from Illumina (San Diego, CA, USA), ON-TARGETplus SMARTpool siRNA targeting Id1 kit from Dharmacon (Waltham, MA, USA), Silencer siRNA Transfection II kit from Ambion (Carlsbad, CA, USA), Agarose I from Amresco (Solon, OH, USA), RNeasy mini kit from Qiagen (Dusseldorf, Germany), Reverse Transcription System from Promega (Fitchburg, WI, USA), SYBR Premix Ex Taq from Takara (Kyoto, Japan), ABI prism 7300 PCR from ABI (Carlsbad, CA, USA), Amersham ECL plus Western Blotting Detection System from GE Healthcare (Little Chalfont, UK), Pierce BCA Protein Assay Kit from Thermo Scientific (Waltham, MA, USA), 8453 UV-visible Spectroscopy System from Agilent (Santa Clara, CA, USA), inverted fluorescence microscope IX51 from Olympus (Tokyo, Japan), and Id1 antibody from Epitomics (Burlingame, CA, USA).

### CCK-8 experiment

Poorly differentiated AGS cells were cultured in Ham’s F12 medium + 10% FBS for 24 h and divided into 7 groups (3 holes in each group). The medium in the holes was substituted with complete medium with final concentrations of 0, 20, 40, 60, 80, 120 and 240 *μ* g/ml ESPS. The proliferation inhibition rate was calculated as follows: (control − administration)/(control − background) × 100%. Control: cells, culture medium, DMSO and CCK-8; administration: cells, culture medium and different concentrations of EGCG and CCK-8; background: only medium and CCK-8.

To verify Id1 gene and protein expression in AGS gastric cancer cells treated with EGCG and siRNA-Id1, 1×10 ^6^ cells were incubated in 12 100-mm culture dishes containing 10 ml complete medium. The medium in 6 culture dishes was substituted with complete medium containing 0, 20, 40, 60, 80 or 100 *μ* g/ml EGCG, and the medium in another 6 culture dishes was substituted with medium containing 0, 40, 60, 80 or 100 nM siRNA-Id1, and 100 nM control siRNA. The complete medium was incubated in 5% CO _2_ at 37°C for 72 h, followed by addition of CCK-8 solution in the proportion 10 *μ* l/100 *μ* l, which was allowed to stand at 37°C for 1 h. Absorbance was read at 450 nm wavelength with a microplate reader.

### Gene expression microarray

AGS cells (3×10 ^6^ ) were incubated in 3 100-mm culture dishes containing 10 ml complete medium. Twenty-four hours later, when all the cells were normal, the medium was substituted with complete medium containing 80 *μ* g/ml EGCG. All treatments were carried out in triplicate. Cells were collected to separate RNA, and total RNA in all samples were extracted and inspected for quality in accordance with Qiagen kit instructions. Qualified total RNA was labeled fluorescently using the Ambion Illumina RNA amplification kit from Illumina, and cRNA was hybridized with Illumina BeadChip HumanHT-12_V4 chip after linear amplification. The film was developed, the hybridization results were scanned, and the relevant data were analyzed and normalized by the Average method. The screening criteria for the differentially expressed genes were as follows: any effective gene either in the experiment group or control group (P<0.05) with the diffscore value in experiment group <13 or >13, and the fold-change >2 of the difference.

### Knockdown of Id1 transcripts using siRNA transfection

We used ON-TARGETplus SMARTpool siRNA targeting Id1 (50 nM, L-005051-00-0050, Dharmacon) and Silencer siRNA Transfection II kit (Ambion) in KO-DMEM medium, according to the manufacturer’s instructions. Non-targeting siRNA (Ambion) was used as a negative control. The ON-TARGETplus SMARTpool siRNA reagent and at least 3 of 4 individual siRNAs silenced target gene expression by at least 75% at the mRNA level when used under optimal delivery conditions (confirmed using a validated control siRNA). Silencing was monitored at the mRNA level 24–48 h after transfection using 100 nM siRNA.

### Apoptosis and cell cycle analysis with flow cytometry

AGS cells were digested with trypsin-EDTA into single-cell suspensions and then collected. The resuspended cells (1×10 ^5^ ) were centrifuged at 1,000 rpm for 5 min to remove the supernatant, and the cells were resuspended in 100 *μ* l Annexin V binding solution and transferred into a 5-ml culture tube. Annexin V-FITC and propidium iodide (PI) (5 *μ* l) was added to the solution, and incubated in the dark at 20–25°C for 15 min, followed by addition of 400 *μ* l Annexin V binding solution for flow cytometry. Annexin V-FITC has green fluorescence and PI has red fluorescence. The wavelength of light excited by the flow cytometer was 488 nm. FITC fluorescence was detected with a band-pass filter of 515 nm wavelength and PI fluorescence was detected with a filter of >560 nm. The cell pellet was added to 1 ml precooled 70% ethanol, fixed at 4°C overnight, washed with PBS twice, centrifuged at 1,000 rpm for 5 min, resuspended in 0.5 ml PBS containing 50 *μ* g/ml PI and 100 *μ* g/ml RNase A, and incubated in the dark at 37°C for 30 min to determine the cell cycle with a flow cytometer according to standard procedures. The result was analyzed with a cycle meter and ModFit software.

### Real-time RT-PCR

Real-time RT-PCR was carried out after treatment of AGS cells. Total RNA was extracted from all samples, quantified, and reverse transcribed into cDNAs according to the instructions of the RNeasy mini kit (Qiagen). Real-time RT-PCR was carried out according to the instructions of the kit of the Reverse Transcription System (Promega). Target gene primer sequences are shown in Table II . Reaction conditions were as follows: 95°C for 30 sec; 95°C for 15 sec, and 62°C for 34 sec (40 cycles). We used the 2 ^−ΔΔ^ Ct method for calculating the relative expression levels of target genes.

### Western blot analysis

Western blot analysis was carried out after drug treatment. The cells were incubated with 5% CO _2_ at 37°C for 48 h, collected after digestion with pancreatin, washed twice with PBS, centrifuged at 2,000 rpm for 5 min to remove the supernatant, and placed on ice for lysis. The proteins were quantified by the bicinchoninic acid (BCA) method. SDS-PAGE, membrane transfer, immunoreactivity and gel electrophoresis image analysis were performed for the target genes.

### Statistical analysis

The data were analyzed with SPSS 13.0 statistical software (SPSS Inc., Chicago, IL, USA), and expressed as the mean ± SD. Multiple groups were analyzed with one-way analysis of variance, pairwise comparison was conducted by using the least significant difference (LSD) t-test, and different groups were compared using a t-test. P<0.05 was statistically significant.

## Results

### Inhibition of AGS cell proliferation and growth by EGCG

**Figure 1.**
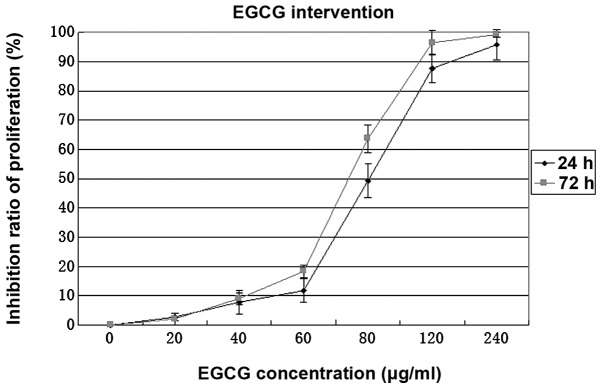
The CCK-8 experiment showed the effect of different concentrations of EGCG on inhibition of AGS cell proliferation. Inhibition rates with different concentrations of EGCG were significantly different (P<0.01). EGCG inhibited AGS cell proliferation in a concentration-dependent manner. Proliferation was significantly inhibited by 80 *μ* g/ml EGCG, and the rates of inhibition of cells treated with the same concentration of EGCG for 24 and 72 h were not significantly different (P>0.05).

The CCK-8 experiment and cell morphological observation showed that EGCG inhibited proliferation of human gastric cancer cells in a concentration-dependent manner ( Fig. 1 , P<0.01). Proliferation of AGS cells treated with 80 *μ* g/ml EGCG was significantly inhibited and the inhibition rate did not differ significantly after 24 and 72 h treatment ( Fig. 1 , P>0.05).

### Selection of differentially expressed genes with gene expression microarray

There were 54 differentially expressed genes when comparing EGCG-treated and normal control cells, with 37 upregulated genes of diffscore value >13 and 17 downregulated genes with diffscore value <−13 ( Table I ). One differentially expressed gene, Id1, in AGS gastric cancer cells before and after treatment with EGCG was screened out, and was verified subsequently ( Table I ). The genes were differentially expressed before and after the AGS gastric cancer cells were treated with EGCG.

### Verification of microarray results with quantitative real-time RT-PCR and western blot analysis

**Figure 2.**
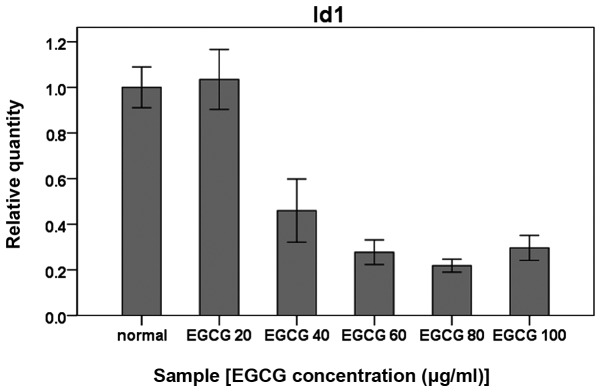
Gene expression microarray analysis of real-time RT-PCR showed that Id1 mRNA expression was significantly reduced in a concentration-dependent manner by EGCG. Id1 mRNA expression in AGS cells was significantly reduced after treatment with 80 *μ* g/ml EGCG.

**Figure 3.**
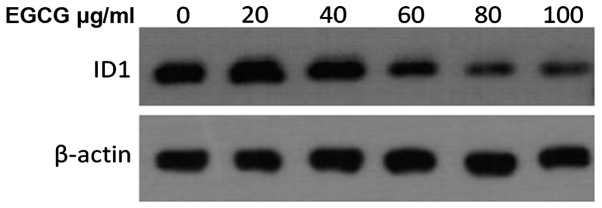
Western blot analysis showed that Id1 protein expression was significantly reduced in AGS cells treated with EGCG in a concentration-dependent manner. Id1 protein expression was significantly reduced after treatment with 80 *μ* g/ml EGCG. β-actin was used as a loading control.

To verify the results from gene expression microarray, the differentially expressed gene Id1 was verified by quantitative real-time RT-PCR, using the primers listed in Table II . Id1 mRNA expression was significantly reduced in the AGS cells treated with EGCG in a concentration-dependent manner ( Fig. 2 , P<0.01). Western blot analysis further showed that Id1 protein expression in AGS cells was consistent with the mRNA expression ( Fig. 3 ).

### Detection of Id1 with CCK-8 to observe influence of RNAi on AGS cell proliferation

**Figure 4.**
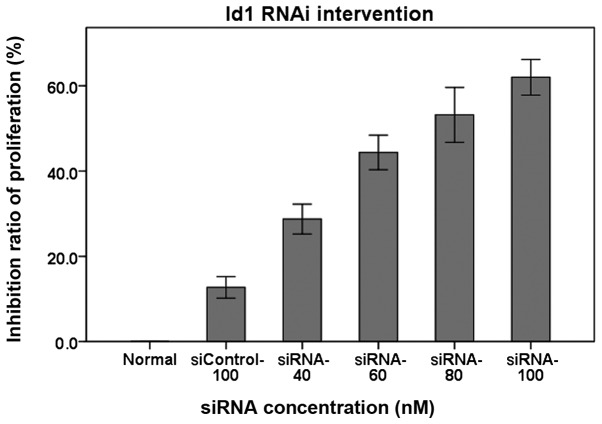
The CCK-8 experiment showed that Id1 RNAi affected AGS cell proliferation. The inhibition rates for cells treated with siRNA-Id1 at different concentrations and 100 nM siRNA controls were significantly different (P<0.01). siRNA-Id1 inhibited proliferation of human AGS gastric cancer cells in a concentration-dependent manner. Proliferation of AGS cells treated with 80 nM siRNA-Id1 was significantly inhibited, but there was no difference between cells treated with the same concentration of EGCG for 24 and 72 h (P>0.05).

The CCK-8 experiment and cell morphological observation showed that siRNA-Id1 inhibited proliferation of AGS cells in a concentration-dependent manner ( Fig. 4 , P<0.01). Proliferation of AGS cells was significantly inhibited by 80 nM siRNA-Id1.

### Id1 expression in AGS cells treated with Id1 RNAi

**Figure 5.**
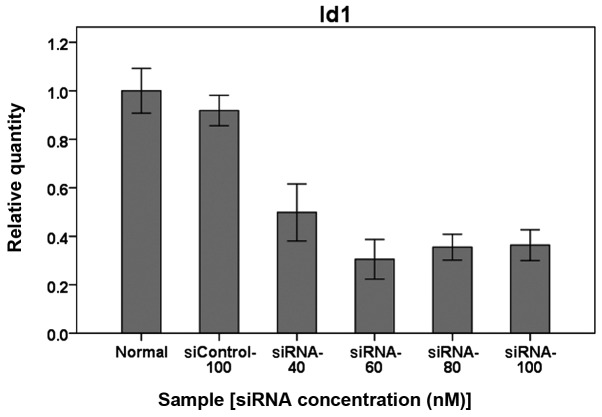
Real-time RT-PCR showed that Id1 mRNA expression in AGS cells treated with siRNA-Id1 was significantly reduced in a concentration-dependent manner. Id1 mRNA expression in AGS cells was significantly reduced after treatment with 80 *μ* g/ml EGCG.

**Figure 6.**
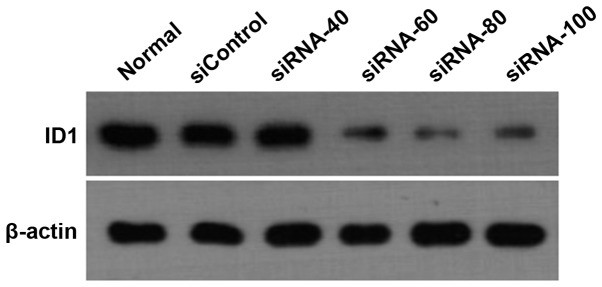
Western blot analysis showed that Id1 protein expression was significantly reduced in AGS cells treated with siRNA-Id1 in a concentration-dependent manner. β-actin was used as a loading control.

Real-time RT-PCR showed that mRNA expression of Id1 was significantly downregulated in AGS cells treated with siRNA-Id1 in a concentration-dependent manner ( Fig. 5 , P<0.01). Western blot analysis further showed that Id1 protein expression in AGS cells was consistent with mRNA expression ( Fig. 6 ).

### Apoptosis and cell cycle of AGS gastric cancer cells treated with EGCG and Id1 RNAi

**Figure 7.**
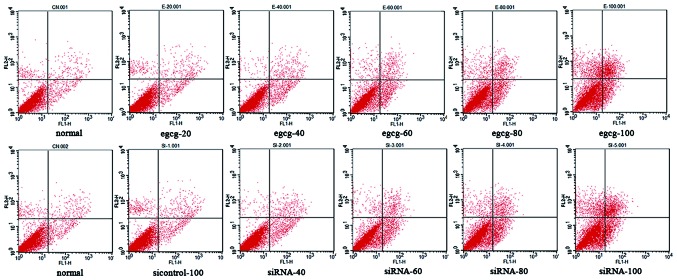
Effect of EGCG and Id1 RNAi on AGS gastric cancer cell apoptosis. Flow cytometry showed that EGCG and siRNA-Id1 concentration-dependently induced apoptosis of AGS cells. Apoptosis and necrosis gradually increased in AGS cells treated with different concentrations of EGCG. Similarly, apoptosis and necrosis also increased gradually in AGS cells treated with different concentrations of siRNA-Id1. No significant difference was found in the two groups for corresponding concentrations of EGCG and siRNA-Id1 (P>0.05). After treatment with Id1-RNAi, proliferation and apoptosis of AGS cells were similar to cells treated with EGCG.

**Figure 8.**
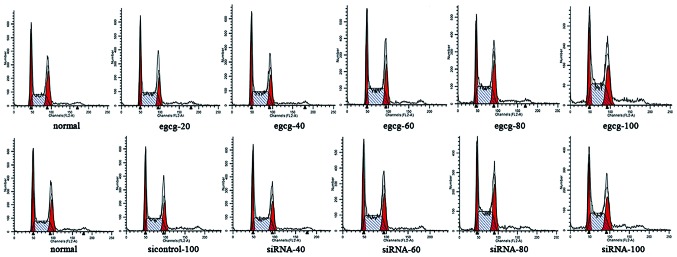
EGCG and Id1 RNAi affected the AGS cell cycle. AGS cells treated with EGCG and siRNA-Id1 were both arrested at S phase (P<0.05). Proliferation and apoptosis of AGS cells treated with Id1-RNAi were similar to the changes in cells treated with EGCG.

Flow cytometry showed that EGCG and siRNA-Id1 induced apoptosis of AGS cells in a concentration-dependent manner ( Fig. 7 ). After treatment with Id1-RNAi, changes in AGS cell proliferation and apoptosis were similar to those in cells treated with EGCG. AGS cells were arrested at S phase after treatment with EGCG and siRNA-ID1 ( Fig. 8 , P<0.05).

## Discussion

An increasing number of studies have shown that EGCG regulates transduction of signaling molecules related to tumor cell metastasis and migration, and inhibits tumor cell proliferation and induces apoptosis and cell cycle arrest ( 13 , 14 ) . In the present study, CCK-8 experiments and morphological observation showed that EGCG inhibited proliferation of AGS gastric cancer cells in a concentration-dependent manner. Flow cytometry showed that EGCG concentration-dependently induced apoptosis of AGS cells and cell cycle arrest at S phase. We further used gene expression microarray analysis for initial screening of differentially expressed genes. There were 54 differentially expressed genes when comparing EGCG-treated and normal control cells, with 37 upregulated and 17 down-regulated genes. Han *et al* have demonstrated that Id1 expression is often high in gastric cancer tissues and cell lines and its expression level is related to the degree of malignancy ( 15 ) . Therefore, high Id1 expression is directly related to the malignant potential of gastric cancer cells. Tsuchiya *et al* have also found that gastric cancer cells with high Id1 expression have strong metastatic ability ( 16 ) . As far as we know, the role of Id1 in EGCG-induced tumor inhibition has not been reported so far. Thus, we determined the differentially expressed Id1 gene by screening with gene expression microarray.

The Id protein family is a helix-loop-helix (HLH) family of transcription factors, including Id1-Id4. Id protein family members have a highly conserved HLH area, which can be combined with a basic HLH protein (bHLH) to form a heterodimer, thereby inhibiting the bHLH binding to target genes, reducing bHLH transcription factor activity, inhibiting cell differentiation and promoting cell proliferation ( 17 , 18 ) . Cell apoptosis and proliferation play an important role in the occurrence and development of malignant tumors ( 19 ) . Id molecules can regulate cyclin-dependent kinase (cdk) inhibitors to shorten the cell cycle. Idl is directly involved in regulating the cell cycle by antisense oligonucleotides ( 20 , 21 ) or microinjection of Id1 antibody ( 22 ) to inhibit partially Id1 protein expression, thus delaying cell entry into S phase. Idl promotes cdk4 and cdk2 to combine with retinoblastoma protein (pRb), by inhibiting transcriptional level of p16 ^ink4a^ and p21 ^WAF1^ , which catalyzes phosphorylation of pRb. E2F and other types of protein can escape from pRb to activate more cancer gene transcription, so the cells enter into S phase and become cancerous ( 23 ) and proliferate ( 24 ) . In fibroblasts, Id1 activates the Ras-Raf-MEK pathway to promote cell proliferation ( 25 ) . Ling *et al* ( 26 ) found that Id1 induces proliferation of prostate cancer cells by activating the mitogen-activated protein kinase pathway, which has a positive correlation with the degree of tumor cell malignancy ( 27 ) . Tumor necrosis factor-α-induced Id1 increases the activity of nuclear factor (NF)-κB and the anti-apoptotic effectors Bcl-xL and intercellular adhesion molecule-1, by inactivating the Bax and caspase-3 pathways, thus inhibiting apoptosis ( 28 ) . Id-1 can also promote Bcl-2 expression and decrease expression of Bax and caspase-3 by inhibiting the p53 signaling pathway and activating the NF-κB signaling pathway, thus preventing tumor cell apoptosis ( 29 ) . Tsuchiya *et al* ( 16 ) reported that tumor cell proliferation and migration are significantly reduced in the Id1/Id3 double knockout gastric cancer cell line MKN 45. The number of peritoneal metastases is significantly reduced, and the size of single metastases is also decreased, which proves that Id1 and Id3 knockout can significantly inhibit peritoneal metastasis of gastric cancer cells. Therefore, Id might be a good target for treatment and prevention of gastric cancer.

After poorly differentiated AGS gastric cancer cells were treated with siRNA-Id1, proliferation slowed significantly, thus inhibiting Id1 mRNA and protein expression. Flow cytometry showed that apoptosis was increased and cells were arrested at S phase. Id1 RNAi affected the phenotypic changes in AGS gastric cancer cells in a dose-dependent manner. The above results suggest that Id1 plays its oncogenic role through promoting cancer cell proliferation and inhibiting apoptosis. Id1 participated in proliferation, apoptosis and other biological behavior of poorly differentiated AGS gastric cancer cells.

Previous studies have shown that EGCG can induce apoptosis through mitochondria ( 30 , 31 ) , p53 ( 32 – 34 ) , Bcl-2 ( 35 ) cell signaling pathways ( 36 , 37 ) , reactive oxygen species ( 38 ) and telomerase ( 39 ) . In different cell lines, EGCG can induce apoptosis through different mechanisms. We found that EGCG lowered Id1 expression to induce apoptosis and inhibit proliferation of poorly differentiated AGS gastric cancer cells, but it is necessary to investigate further how to achieve the above through a certain mechanism and whether the activation of signal transduction pathways is affected.

Molecular target therapy has become the trend in cancer treatment and research due to its high selectivity, good efficacy and few side-effects. Our experiments proved that EGCG could play a role in inhibiting proliferation, promoting apoptosis, and affecting cell cycle of poorly differentiated AGS gastric cancer cells, which is closely related to down-regulation of Id1 expression. Therefore, Id1 may be one of the targets regulated by EGCG for tumor inhibition.

## Figures and Tables

**Table I.** cDNA microarray expression profiling of differentially expressed genes.

Probe ID	Symbol	EGCG. AVG signal	EGCG. detection p-value	EGCG. diffscore	EGCG vs CN fold-change	CN.AVG signal	CN. detection p-value	Search key	Definition
ILMN_1689842	SC4MOL	469.9426	0	44.64191	2.146925	218.891	0	NM_006745.3	Human sterol-C4-methyl oxidase-like (SC4MOL), transcript variant 1, mRNA
ILMN_1664861	ID1	1,280.916	0	−40.1308	0.483405	2649.78	0	NM_181353.1	Human Id1, dominant negative HLH protein (ID1), transcript variant 2, mRNA
ILMN_1732296	ID3	390.6099	0	−47.3221	0.467825	834.9481	0	NM_002167.2	Human Id3, dominant negative HLH protein (ID3), mRNA

There were 54 differentially expression genes, including 37 upregulated genes of diffscore value >13 and 17 downregulated genes of diffscore value <−13 after comparing the EGCG-treated and normal control groups. The table shows three differentially expressed genes by cDNA microarray expression profiling screening, including a significantly upregulated gene: SC4MOL, and two significantly downregulated genes: Id1 and Id3.

**Table I.** Real-time RT-PCR primers.

SN	Gene	Primer sequence (5′ to 3′)
1	β-actin	F: 5′TGGAGAAAATCTGGCACCA3′ R: 5′CAGGCGTACAGGGATAGCAC3′
2	Id1	F: 5′ACGACATGAACGGCTGTTACTCAC3′ R: 5′CTCCAACTGAAGGTCCCTGATGTAG3′

## Abbreviations:

EGCG: epigallocatechin-3-gallate;
CCK-8: cell counting kit-8;
PI: propidium iodide
